# Interphase Engineering Enabled by Using a Separator with Electrochemically Active Carbazole Polymers for Lithium-Ion Batteries

**DOI:** 10.3390/polym17131815

**Published:** 2025-06-29

**Authors:** Bingning Wang, Lihong Gao, Zhenzhen Yang, Xianyang Wu, Qijia Zhu, Qian Liu, Fulya Dogan, Yang Qin, Chen Liao

**Affiliations:** 1 Chemical Sciences and Engineering Division, Argonne National Laboratory, 9700 South Cass Avenue, Lemont, IL 60439, USAyangzhzh@anl.gov (Z.Y.); xianyang.wu@anl.gov (X.W.); zhuq@anl.gov (Q.Z.); qian.liu@anl.gov (Q.L.); fdogan@anl.gov (F.D.); 2 Department of Chemical & Biomolecular Engineering, Institute of Materials Science, University of Connecticut, Storrs, CT 06269, USA; yang.qin@uconn.edu; 3 Energy Storage Research Alliance, Argonne National Laboratory, 9700 South Cass Avenue, Lemont, IL 60439, USA

**Keywords:** separator modification, lithium-manganese-rich, carbazole, polymer oxidation, full cells, lithium-ion batteries

## Abstract

Separators are generally considered inert components in lithium-ion batteries. In the past, some electroactive polymers have been successfully applied in separator modifications for overcharge protection or as acid scavengers. This study highlights the first use of two “electroactive” carbazole polymers (copolymer 9-phenyl-9H-carbazole-phenyl [PCP] and poly(9-vinylcarbazole) [PVC]), which were each applied separately as coatings on the cathode-facing side of commercial Celgard 2325 separators, respectively, to enhance the cycling performance of 0.3Li_2_MnO_3_·0.7LiMn_0.5_Ni_0.5_O_2_//graphite (LMR-NM//Gr) full cells through interphase engineering. The team observed an irreversible polymer oxidation process of the carbazole-functionalized polymers—occurring only during the first charge—for the modified separator cells, and the results were confirmed by *dQ*/*dV* analysis, cyclic voltammetry measurements, and nuclear magnetic resonance characterizations. During this oxidation, carbazole polymers participate in the process of interphase formation, contributing to the improved cycling performance of LMR-NM//Gr batteries. Particularly, oxidation takes place at voltages of ~4.0 and ~3.5 V when PCP and PVC are used as separator coatings, which is highly irreversible. Further postmortem examinations suggest that the improvements using these modified separators arise from the formation of higher-quality and more inorganic SEI, as well as the beneficial CEI enriched in LixPOyFz. These interphases effectively inhibit the crosstalk effect by reducing TM dissolution.

## 1. Introduction

0.3Li_2_MnO_3_·0.7LiMn_0.5_Ni_0.5_O_2_(LMR-NM) is a promising cobalt-free, lithium- and manganese-rich cathode material for next-generation, high-energy-density lithium-ion batteries (LIBs), addressing concerns related to driving range anxiety and supply chain sustainability [1,2,3]. However, it suffers from severe capacity loss and voltage fade during cycling, making it challenging to achieve long-term cycling stability for practical applications [4]. Numerous efforts ranging from cathode synthesis [5,6], element doping [7], surface coating [8], and electrolyte optimization [8,9] have been developed to handle these issues. Another innovative approach involves using carbon nanostructures for anodes (nanostructured carbon) [10] and cathodes (carbon coating) [11], which can also enhance energy density. Each method has its own limitations, and a combinatorial approach encompassing multiple aspects is necessary. Cathode synthesis primarily focuses on cathode development, including elemental doping. However, the energy density and voltage fade of these cathodes can still pose challenges. Other methods, such as surface coating and electrolyte optimization, are highly dependent on the specific cathode material. Notably, the trend of using AI [8] or machine learning approaches is becoming increasingly popular. Enhancing control and performance through separator modifications could further improve energy density.

In this study, we present a new approach using separator modification to enhance the cycling performance of LIBs, given that polymers offer such advantages as easy modification for specific purposes featuring tunable functional groups and limited solubility in solvents [12,13]. The development of separators is vital for battery performance improvement [14]; from the point of view of materials properties, the characteristics that are the most important include the MacMullin number [15], Gurley number [15], air permeability, thickness, porosity/pore size, wettability, contact angle test, chemical stability, thermal lateral shrinkage, and shutdown temperature [16]. Typically, separators are considered to be electrochemically inert components in LIBs, primarily functioning as electric insulators between the cathode and the anode and serving as electrolyte reservoirs [17,18]. Although electroactive polymers have been applied in separator modifications, they are generally used for overcharge protection [19,20,21,22,23] or for scavenging undesired acidic decomposition [24]. In contrast, to the best of our knowledge, this is the first time that polymers have been introduced into separator modifications as an “electroactive” component to enhance the electrochemical performance of lithium-ion batteries by participating in interphase formation.

**Figure 1:**
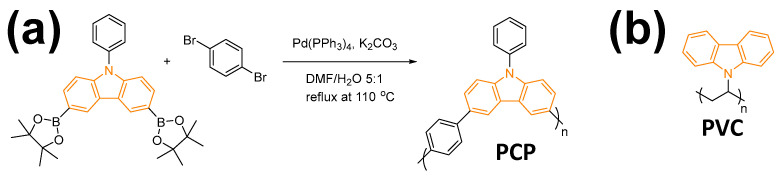
(**a**) Synthesis and molecular structure of copolymer of 9-phenyl-9H-carbazole-phenyl (PCP), (**b**) molecular structure of poly(9-vinylcarbazole) (PVC).

In this work, two carbazole-functionalized polymers, (1) copolymer of 9-phenyl-9H-carbazole-phenyl (PCP) and (2) poly(9-vinylcarbazole) (PVC), representing two types of polymer structures, were coated on the Celgard 2325 separators—on the side facing the cathode only—using a simple slurry coating method. The PCP, which was synthesized in-house, consisted of a polymer-phenyl-carbazole backbone, while the PVC comprised a polyethylene (PE) backbone with carbazole units as side chains; PVC is commercially available and was used in its original form (Figure 1a,b). As will be addressed in the following discussion, the carbazole functional groups undergo irreversible oxidation during the first charge, facilitating the formation of a higher-quality and more inorganic solid electrolyte interphase (SEI) [25,26,27], as well as a beneficial cathode electrolyte interphase (CEI) enriched in lithium oxyfluorophosphates (LixPOyFz) [28]. Through an array of experiments, we demonstrate that these interphases (1) effectively inhibit the crosstalk effect by reducing transition metal (TM) dissolution, (2) significantly enhance the cycling stability, and (3) also increase the lithium inventory and Coulombic efficiency (CE) of LMR-NM//graphite (Gr) full cells through the oxidation of the polymers.

## 2. Materials and Methods

### 2.1. Synthesis of PCP

The copolymer of 9-phenyl-9H-carbazole-phenyl (PCP) was synthesized via a Suzuki coupling reaction. Then, 1 mmol of 9-Phenyl-3,6-bis(4,4,5,5-tetramethyl-1,3,2-dioxaborolan-2-yl)-9H-carbazole (TCI America, 9211 North Harborgate Street, Portland, OR 97203, USA), 1 mmol of 1,4-dibromobenzene (Sigma-Aldrich, 3050 Spruce St, Saint Louis, MO 63103, USA), 6 mmol of K_2_CO_3_ (Sigma-Aldrich), and 0.1 mmol of Pd(PPh_3_)_4_ (Sigma-Aldrich) were added to a two-neck round-bottom flask with 50 mL of solvent of dimethylformamide/deionized water at a volume ratio of 5:1. The reactants were then subjected to reflux with stirring for 72 h at 110 °C under a nitrogen atmosphere. After the reaction, the mixture was filtered. The collected solids were then poured into dimethylformamide and washed at 50 °C overnight to remove the residues of the reactants. After filtration, the solids were further washed with deionized water and then dried under vacuum at 60 °C.

### 2.2. Separators Coating

The synthesized PCP and commercially available poly(9-vinylcarbazole) (PVC) (Sigma-Aldrich) were used in the separators coating. The carbazole functionalized polymers and polyvinylidene fluoride (PVDF) were well mixed to form a uniform slurry at a ratio of 7:3 with a proper amount of N-Methyl-2-pyrrolidone (NMP), and the PVDF was from a solution of 8 wt.% PVDF in NMP. Then, the slurry was coated on the Celgard 2325 separators (Celgard, LLC, 13800 South Lakes Drive, Charlotte, NC 28273, USA) manually using a doctor blade (with a thickness of 50, 100, or 150 μm). The separators were dried in a vacuum oven overnight at 45 °C to remove NMP, cut into 16 mm diameter pieces, and then further vacuum dried in an Ar-filled glovebox at 45 °C before use.

### 2.3. Electrochemical Test

Cell cycling tests were performed in full cells of LMR-NM//Gr with 20 μL of the Gen2 electrolyte at room temperature. Celgard 2325 separators were used as the baseline. The test protocol [29] included three formation cycles at a C/10 rate (1 C = 2.8 mA) with a voltage window of 2.5–4.3 V, followed by two activation cycles at C/20 with a voltage window of 2.5–4.6 V, and 80 aging cycles at C/3 with a voltage window of 2.5–4.4 V. Right before and after every 20 aging cycles, the cell performance was checked with a slow C/25 cycle with a voltage window of 2.5–4.4 V to test its cycling performance at a slow rate, a fast 1 C cycle with a voltage window of 2.5–4.4 V to test its cycling performance at a high rate, and a modified hybrid pulse power characterization (HPPC) sequence to examine its impedance. The LMR-NM cathode consisted of 84 wt.% of active material, 8 wt.% of Timcal C45 (US Imerys Graphite & Carbon USA Inc., 29299 Clemens Road, Unit 1-L, Westlake, OH 44145, USA) carbon, and 8 wt.% Solvay 5130 PVDF binder (4500 McGinnis Ferry Road, Alpharetta, GA 30005, USA), with a mass loading of 11.15 mg/cm^2^. The Gr anode consisted of 91.83 wt.% of Superior Graphite SLC 1506T (550 W. Van Buren St., Suite 300, Chicago, IL 60607, USA), 2 wt.% of Timcal C45 carbon, 6 wt.% Kureha 9300 PVDF binder (3151 Briarpark Drive - Suite 1025, Houston, TX 77042, USA), and 0.17 wt.% of oxalic acid (Sigma Aldrich), with a mass loading of 9.96 mg/cm^2^.

Cyclic voltammetry measurements were performed at a scan rate of 0.1 mV/s for PCP//Li cells within a voltage window of 1–5 V, and for PCP//Cu and Al//Cu cells within a voltage window of 0–5 V. The PCP electrodes consisted of 70 wt.% PCP and 30 wt.% PVDF without added carbon, coated on Al foils using a slurry coating method.

### 2.4. Fourier Transform Spectroscopy (FT-IR) Characterization

The reactants of 9-Phenyl-3,6-bis(4,4,5,5-tetramethyl-1,3,2-dioxaborolan-2-yl)-9H-carbazole and 1,4-dibromobenzene and the synthesized product of PCP were subjected to FT-IR characterization using a Thermo Scientific Nicolet iS5 FT-IR Spectrometer (168 Third Avenue, Waltham, MA 02451, USA) over a range of 500 to 4000 cm^−1^. The disappearance of B-O (1346.55 cm^−1^) and C-Br (1064 cm^−1^) in the synthesized PCP confirmed its successful synthesis.

### 2.5. X-Ray Photoelectron Spectra (XPS) Characterization

XPS was performed by using a PHI 5000 VersaProbe II System (Physical Electronics, 18725 Lake Dr E, Chanhassen, MN 55317, USA) with a base pressure of 2 × 10^−9^ Torr. The cycled anodes and cathodes were harvested from the aged cells and carefully washed with DMC prior to measurement. The photoelectron spectra were obtained in the fixed analyzer transmission mode using Al Kα radiation (hν = 1486.6 eV, 100 μm beam, 25 W) with Ar^+^ and electron beam sample neutralization. XPS spectra were aligned to the graphitic carbon at 284.5 eV.

### 2.6. Inductively Coupled Plasma-Mass Spectra (ICP-MS) Characterization

To quantitatively evaluate transition metal dissolution in the aged cells, the cycled anodes were rinsed with DMC, transferred to a quartz beaker, and burned in a furnace at 700 °C for 12 h to remove organic constituents and carbon. The ash was refluxed with a mixture of nitric and hydrochloric acids at 220 °C for 1 h, and the solutions were diluted with water. The samples were analyzed using inductively coupled plasma-mass spectrometry (ICP-MS) to determine the transition metal concentrations that were referred to the weight of the anode. Measurements were made using a PerkinElmer NexION 2000 ICP Mass Spectrometer (940 Winter St, Waltham, MA 02451, USA) calibrated with the NIST traceable standards.

### 2.7. Nuclear Magnetic Resonance (NMR) Spectroscopy

To test the solubility of the carbazole polymers in the Gen2 electrolyte, specific amounts of PCP and PVC were dispersed in Gen2 and stirred vigorously for 2 days, respectively. After stirring, the electrolytes were filtered with a 0.22 μm pore size syringe and then injected into NMR tubes. Fluorinated ethylene propylene (FEP) NMR tube liners filled with benzene-d_6_ were included in the NMR tubes as a reference. The Gen2 electrolytes were then subjected to ^1^H NMR analyses to check if any PCP or PVC had dissolved.

To verify the oxidation of PCP, a PCP//Li half-cell was assembled, charged to 5 V with a current of 10 μA, and then disassembled. The PCP electrode was subsequently immersed in dimethyl sulfoxide-d_6_ (DMSO-d_6_), which dissolved easily due to the increased polarization caused by the oxidation of the PCP. The resulting solution was then transferred into an NMR tube. A fluorinated ethylene propylene (FEP) NMR tube liner filled with LiPF_6_ DMSO-d_6_ solution was also included as a reference. The solution was then subjected to ^19^F and ^31^P NMR analyses. For comparison, PCP polymers were also dispersed in DMSO-d_6_ and sonicated for 2 h, but no dissolution was observed.

### 2.8. Electrolyte Uptake

Separators were weighed before and after being totally immersed in Gen2 electrolyte. After complete wetting, excess electrolyte attached to the separator surfaces was removed by filter papers. The electrolyte uptake for each separator was then calculated by the equation:

Electrolyte uptake = (W_wet_ − W_dry_)/W_dry_ × 100%, (1)

where W_dry_ and W_wet_ are the weights of separators before and after wetting by Gen2, respectively.

## 3. Results and Discussion

PCP was synthesized via Suzuki coupling, as presented in Figure 1a and confirmed by Fourier transform infrared (FT-IR) analysis (see Figure S1). PCP was applied to modify Celgard 2325 separators using the slurry coating method on the side facing the cathode (see Figure S2). Slurries of 70 wt.% carbazole polymers and 30 wt.% polyvinylidene fluoride (PVDF) binders were well dispersed in proper amounts of N-methyl pyrrolidone (NMP) solvent. The slurries were then coated on commercial separators with a doctor’s blade, with blade thicknesses of 50, 100, or 150 μm. After coating, the separators were dried under vacuum at elevated temperatures to remove the NMP solvent, and the mixture of carbazole polymers and PVDF binders was left on the separator as the coating layers. The thicknesses of the modified separator ranged between 47 and 70 µm.

**Figure 2:**
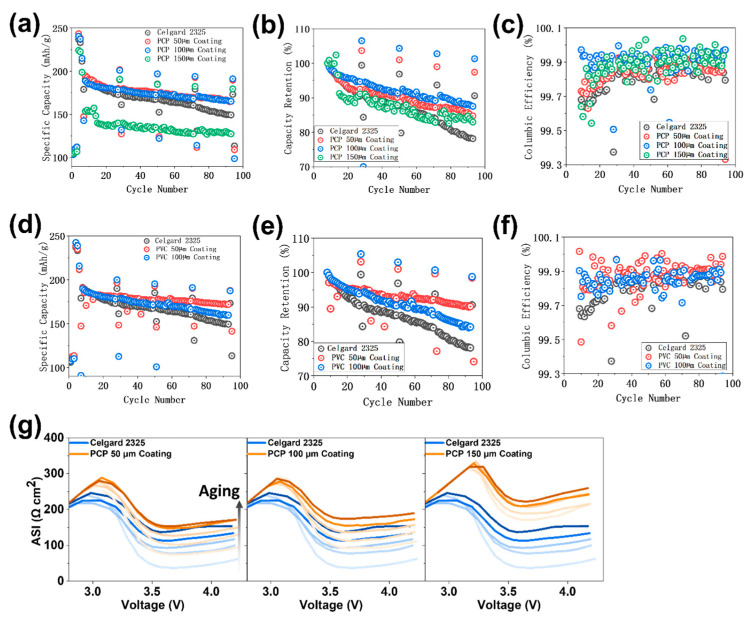
Cycling-specific capacities (**a**,**e**), capacity retentions (**b**,**f**), and Coulombic efficiencies (**c**,**g**) for LMR-NM//Gr full cells using PCP and PVC, respectively, modified Celgard 2325 separators with different coating thicknesses compared to the cell using baseline Celgard 2325 separator. (**d**) Area-specific impedance (ASI) for LMR-NM//Gr cell using different thicknesses PCP coating separators compared to the baseline cell using Celgard 2325. The darker lines are the more aged cycles.

The performance of the modified separators was evaluated using LMR-NM//Gr full cells. The cycling results, including specific capacities, capacity retentions, and CEs, are presented in Figure 2a–c, respectively. Separators with 50- and 100-μm PCP coating exhibit improved cycling performance compared to the Celgard 2325 baseline (see Figure 2a). The separator with a 150-μm coating stabilizes after ~10 cycles but overall shows decreased cycling-specific capacities.

As depicted in Figure 2b, PCP modifications improve the cells capacity retentions of the C/3 aging cycles (compared with cycle 8, the first C/3 aging cycle) from 78.13% of the baseline cell to 84.72%, 87.48%, and 82.94% for coating thicknesses of 50, 100, and 150 μm, respectively. This result highlights the significant benefits of making PCP modifications in enhancing the cells’ durable cycling. Additionally, PCP modifications improve the cells’ levels of CE (see Figure 2c), indicating that side reactions are suppressed [30]. The 100-μm PCP coating demonstrates an average CE of 99.92%, higher than the baseline cell’s 99.81%.

The observed benefits are attributed to the carbazole functional groups of PCP, given the stability and inertness of the benzene rings present in the main backbones of PCP. To further explore the potential of the carbazole functional group in enhancing cells’ performance via separator modification, PVC, a commercially available polymer with carbazole functionality and a PE backbone, was also examined using the same coating method at different thicknesses. Its electrochemical cycling results are presented in Figure 2d–f. We tested only the 50- and 100-μm coating thicknesses for PVC, as the 150-μm coating led to the cells’ failure at initial cycles in our experiments. Improved cycling-specific capacities, capacity retention levels, and CE levels were observed for LMR-NM//Gr full cells using the PVC-coated separators. Particularly, in the case of the 50-μm-thick coating, the capacity retention and average CE of the C/3 aging cycles increase from 78.13% and 99.81% of the baseline cell to 90.14% and 99.88%, respectively.

Although both PCP- and PVC-coated separators demonstrate impressive improvements in cycling stability and CE, the impedances of the cells using modified separators, particularly the initial impedances, increase noticeably, as presented in Figure 2g, Table S1, Figure S3, and Table S2. The initial area-specific impedances (ASIs) for 50-, 100-, and 150-μm PCP coatings increase from 37.63 Ω cm^2^ for the baseline cell to 74.37, 92.84, and 170.97 Ω cm^2^, respectively. Similarly, thicker PVC coatings result in higher initial impedances. However, for the 50-μm PVC coating, the final impedance decreases to 76.36 Ω cm^2^, which is lower than the baseline cell’s 137.38 Ω cm^2^, showing only a 45.36% increase.

The rise in impedance is attributed to the simple slurry coating method we used. As presented in Table S3, there is a notable decrease in the ability of the modified separators to uptake electrolyte, with this effect becoming more pronounced as the coatings become thicker. No micro- or nano-structures were introduced into the polymers’ slurry coating processes. Consequently, the coating layer blocks the micropores of the separator, impeding Li+ transport channels and compromising the wetting capability, which leads to higher impedances [18,31]. Advanced separator manufacturing techniques may offer solutions to address this issue. Although a thin coating is designed to achieve both high energy density and efficient electrochemical processes, the simple doctor’s blade method we currently use can only produce relatively thick films. It’s important to note that the thickness mentioned refers to the doctor’s blade thickness, not the actual thickness of the dried film, which measures between 47 and 70 µm for PCP- and PVC-polymer-coated separators. Since our primary focus is on the chemistry and electrochemistry aspects, future plans for polymer membrane engineering could significantly reduce the thickness. Various film deposition technologies, such as vapor deposition (including sputtering [32], atomic layer deposition [33], and solid-vapor methods), liquid-phase coating, electrodeposition, plasma polymerization [34], and in-situ polymerization, can be employed. Among these, solution-based deposition offers advantages like high throughput, low capital investment, and compatibility with roll-to-roll (R2R) manufacturing. Additionally, gravure coating [35], which enables the production of highly uniform sub-micron films, presents a viable option for future industrial coating and printing collaborations with industrial partners. The scope of this work primarily focuses on reporting the improved electrochemical performance of LIBs enabled by carbazole polymer-coated separators via interphase engineering, which facilitates the formation of higher-quality SEI and CEI. Table 1 summarizes the comparisons of specific capacity, ASI, and 8th cycle coulombic efficiency for baseline separators and polymer-coated separators.

The research team also examined the solubility of PCP and PVC in Gen2 electrolyte to ensure their suitability as separator components. A substantial amount of each polymer was dispersed in Gen2 and stirred vigorously for two days. The Gen2 electrolytes were then collected and subjected to ^1^H NMR measurements, which showed no peaks from the polymers, confirming the insolubility of both PCP and PVC in Gen2 (see Figure S4).

**Figure 3:**
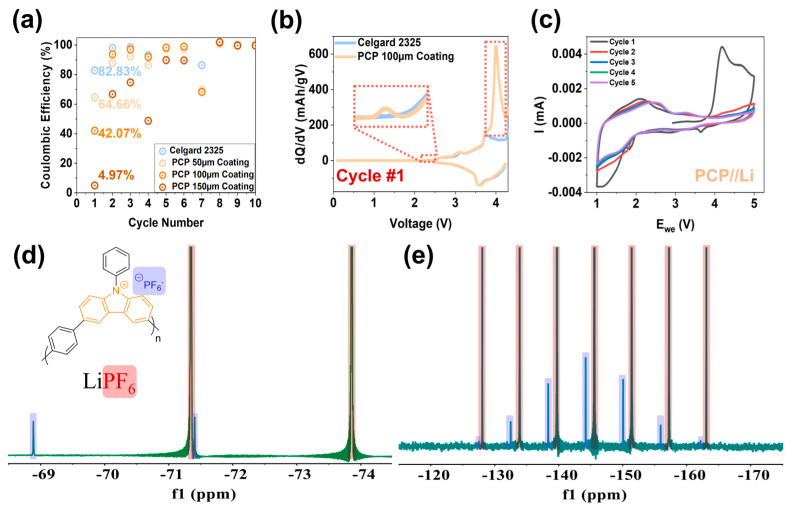
(**a**) Coulombic efficiencies of the initial cycles for LMR-NM//Gr full cells using PCP-modified Celgard 2325 separators with different coating thicknesses compared to the cell using baseline Celgard 2325 separator. The inserted numbers are the initial Coulombic efficiencies for each cell as denoted. (**b**) Differential capacity (*dQ*/*dV*) curves for the PCP 100 μm coated separator cell compared to the baseline cell for cycle 1, the first formation cycle. (**c**) Cyclic voltammetry curves of PCP//Li at a scan rate of 0.1mV/s. (**d**) ^19^F and (**e**) ^31^P NMR spectra of the oxidized PCP dissolved in DMSO-d6. A fluorinated ethylene polypropylene liner filled with LiPF_6_ dissolved in DMSO-d6 was used as the reference. The peaks marked in blue are from the oxidized PCP, while peaks marked in red are from LiPF_6_.

To elucidate the fundamental working mechanisms of the benefits provided by the carbazole polymers, the analyses and discussions focus primarily on the cells modified with PCP as an illustrative example. As presented in Figure 3a, the initial CE levels of the PCP-coated cells are lower than those of the baseline and decrease with increasing coating thickness. However, the differences gradually disappear over cycling without interfering with the electrochemical performances in subsequent cycles. To investigate the underlying reason, the differential capacity (*dQ*/*dV*) profiles of the LMR-NM//Gr cells were plotted. Figure 3b and Figures S5–S7 present the *dQ*/*dV* curves of cycle 1 (the first formation cycle), cycle 2 (the second formation cycle), cycle 4 (the first activation cycle), and cycle 8 (the first C/3 aging cycle) for each modified cell as compared to the non-modified baseline. For the 50- and 100-μm coated cells, during the first charge, two unique peaks at ~2.3 and 4.0 V are observed, whereas in the subsequent discharge process and following cycles, the *dQ*/*dV* profiles match the baseline without presenting any additional peaks. The intensity of the unique peak at 4.0 V during the first charge increases with the PCP coating amounts, indicating a direct correlation between the quantity of PCP coating and the occurrence of additional reactions. This correlation likely explains the observed improvement in electrochemical performance. When the coating thickness increased to 150 μm (see Figure S7), the additional peak at ~4.0 V in the first charge was extremely high and was still noticeable in cycles 2 and 4 due to the excessive PCP coating. However, no extra peaks are present in any discharge processes or in cycle 8. Furthermore, as presented in Figure S8, the charge-specific capacity increases with the thickness of the PCP coating, particularly in the initial cycles. This result suggests that lithium inventory was enhanced due to the irreversible PCP oxidation [36].

**Figure 4:**
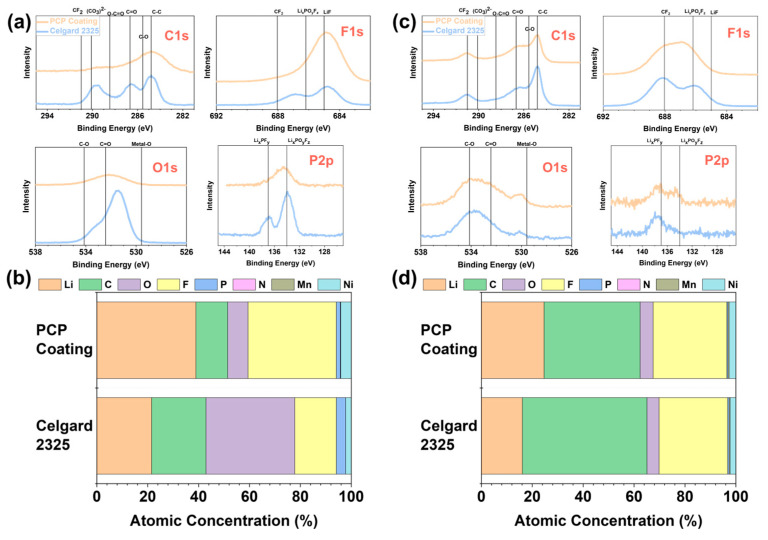
C1s, F1s, O1s, and P2p regions of XPS spectra for the (**a**) anodes and (**c**) cathodes collected from cycled PCP coating cells compared to the baseline. And atomic concentrations for the (**b**) anodes and (**d**) cathodes XPS spectra.

Recent developments in anion-intercalation and dual-ion batteries, which utilize anion storage, are emerging as promising alternatives for current metal-ion batteries due to their high theoretical capacity and sustainability [37,38,39,40,41]. For instance, PF_6_ can intercalate into Gr, forming Gr intercalation compounds at high potentials, around 5 V [42]. Similarly, organic materials such as N-substituted carbazole derivatives and polycyclic aromatic hydrocarbons are engineered as cathode materials owing to their ability to undergo oxidization and facilitate anion intercalation at high voltages [39,41]. In the case of PCP-modified cells, the additional peaks observed in the first charging *dQ*/*dV* curves of PCP-modified cells are attributed to the oxidation of PCP (see Figure 4).

Cyclic voltammetry (CV) analysis was also performed to confirm the oxidation of PCP. Figure 3c presents the CV curves of a PCP//Li half-cell. An oxidation peak at ~4.1 V is observed in the first cycle, corresponding to an additional 4.0 V peak observed in the previous *dQ*/*dV* curves of LMR-NM//Gr cells, implying the oxidation of PCP. Although two weak reduction peaks are observed at 3.6 and 1.8 V, no oxidation or reduction peaks appear in the subsequent scans, indicating that the reversibility of PCP oxidation is very limited and that the PF_6_^−^ intercalated PCP would remain electrochemically inert after the first charge. Figure S9 compares the CV curves of PCP//Cu compared to Al//Cu cells. When PCP was coated on the Al current collector, a pronounced oxidation shoulder between 1.5 and 4.5 V appeared during the first scan. Other peaks overlap well in both cells, attributed to the oxidation and reduction of electrolyte. This result also supports the conclusion that the oxidation of PCP is irreversible.

This PCP oxidation process was further confirmed via ^19^F and ^31^P NMR measurements in a coin cell setup. A laminate with PCP active material was paired up with Li metal and charged to 5 V using Gen2 as an electrolyte. The laminate was then immersed in dimethyl sulfoxide-d_6_ (DMSO-d_6_), and the solution was subsequently subjected to ^19^F and ^31^P NMR measurements. After being charged to 5 V, the oxidized PCP polymer became soluble in DMSO-d_6_ due to the increased polarity (see Figure S10). The resulting ^19^F and ^31^P NMR spectra clearly present the existence of PF_6−_ in the oxidized PCP (see Figure 3d,e). NMR spectroscopy is a common tool for understanding electrolyte changes and decomposition products [43]. In this context, the solubility of the products changes significantly, enabling a more detailed analysis. As illustrated in Figure 3, the ^19^F NMR spectrum of PF_6_^−^ from LiPF_6_ is characterized by a doublet with a chemical shift of −72.6 ppm. In contrast, the charged PCP polymer counter anion PF_6_^−^ exhibits a more downfield shift with a chemical shift of −70.1 ppm. Although changes in the ^31^P NMR spectrum are less pronounced, the septet is still shifted slightly downfield.

To elucidate the mechanism behind the enhanced cycling performance resulting from separator modifications, X-ray photoelectron spectroscopy (XPS) measurements were performed on the collected anodes and cathodes after cycling. Figure 4a presents the C1s, F1s, O1s, and P2p regions of XPS spectra for the cycled anodes collected from the PCP-coated cell and the baseline. The atomic concentrations from XPS measurements of the anode are presented in Figure 4b, with results consistent with the observations from the spectra. SEI, as “the most important and least understood” component of LIBs, has generated numerous studies employing various characterization techniques to investigate its structure, composition, and properties [44]. It is suggested that SEI exhibits a bilayer feature, with an inner layer rich in inorganic compounds overlaid by an outer layer rich in organic compounds [45]. As shown in Figure 4a, the SEI outer layer using a PCP-coated separator was modified to be more inorganic and enriched in LiF crystals (685 eV), which are widely reported to be beneficial protective SEI components for the electrochemical performance of LIBs [26,46,47,48]. The SEI outer layer [49,50] also shows less organic (CO_3_)^2−^ and C=O in the F1s and C1s regions, respectively, and an obvious difference in the P2p region. XPS spectra of the less prominent elements of N1s, Li1s, Mn2p, and Ni2p are shown in Figure S11.

The CEI compositions were also examined using XPS, with the results presented in Figure 4c,d and Figure S12. The F1s and P2p spectra show the most significant differences, revealing that the CEI of the PCP-coated cell is richer in Li_x_PO_y_F_z_, which is widely acknowledged as beneficial in CEI components [28].

Based on the *dQ*/*dV* analyses and XPS results, it can be concluded that, by virtue of the irreversible carbazole polymer oxidation, PCP participates in the interphase formation process, driving the establishment of beneficial interphases on both anode and cathode sides, which results in the improved cycling performance of LMR-NM//Gr full cells. The existence of these oxidized polycarbazole molecules with counter anions has the capability of modifying the interphases. Improved cycling stability could be partially due to the potential thick coating layer and the suppressed crosstalk of the TM species that has been proven to be detrimental to energy density.

**Figure 5:**
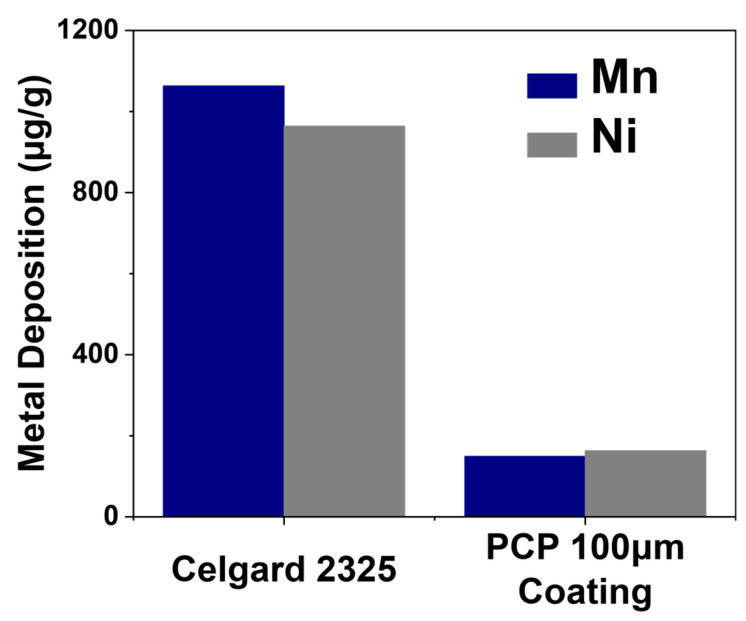
ICP-MS analysis of cycled anodes of PCP 100 μm coated separator, and Celgard 2325 baseline cells.

Because the 100-μm PCP-coated cell exhibits the highest CE among all the PCP coating thicknesses, indicating a significant number of mitigated side reactions during cycling, inductively coupled plasma mass spectrometry (ICP-MS) measurements were conducted for its cycled anode and compared them to the Celgard 2325 baseline to examine the effect of PCP-driven interphases on TM dissolution. As presented in Figure 5, TM dissolutions are significantly reduced for the PCP-modified cell, with Mn dissolution decreasing from 1063.64 μg/g in the baseline cell to 148.63 μg/g. The mitigated TM dissolutions can notably alleviate the crosstalk effect, which is a notorious culprit for cell degradation [51,52], owing to the interphase’s improvement caused by the oxidation of PCP during the first charge.

As with PCP, the PVC-modified cells exhibit decreased initial CEs and increased initial charge capacities (see Figure S13). Additional peaks at ~2.3 and 3.5 V are observed for the PVC-modified cells only in the first charging process (see Figures S14 and S15). This finding indicates that being oxidized is a common feature of carbazole functional groups at high voltages and is irreversible in LMR-NM//Gr full cells cycling. Additionally, the difference in oxidation voltages between PCP and PVC demonstrates that the oxidation potential of carbazole functional groups is influenced by the specific molecular structures surrounding them.

**Figure 6:**
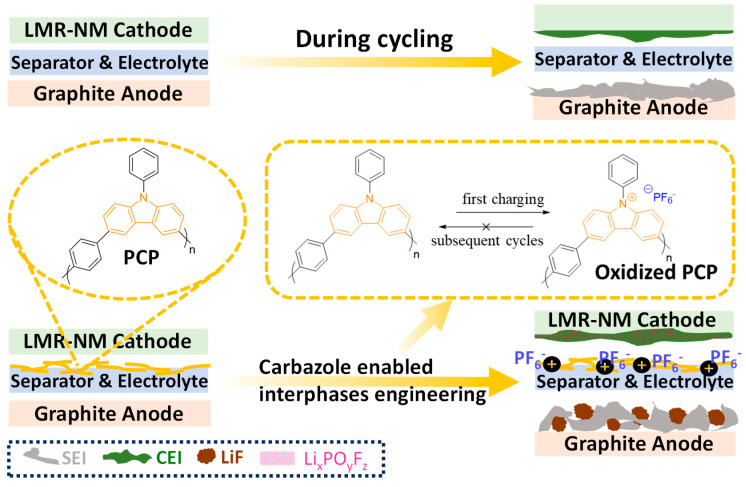
Illustration of the interphases engineering enabled by the polymer oxidation process of PCP as a cathode-side separator coating layer in LMR-NM//Gr cell compared to the non-coated separator.

In summary, as shown in Figure 6, during the first charging of LMR-NM//Gr full cells using the modified separators, irreversible polymer oxidation occurs in the carbazole functionalized polymers, leading to the low initial CEs observed and providing additional lithium inventory and the formation of advantageous interphases. Oxidations take place at voltages of ~4.0 and ~3.5 V when PCP and PVC are used as the separator coating layers, respectively, and are highly irreversible according to *dQ*/*dV* and CV analyses, ensuring that the carbazole polymers are electroactive during the initial charge only and without interfering with subsequent cell cycling. After the cells are tested, postmortem examinations suggest that the improvements using these modified separators arise from the formation of higher-quality and more inorganic SEI as well as the beneficial CEI enriched in Li_x_PO_y_F_z_. These interphases effectively inhibit the crosstalk effect by reducing TM dissolution.

As illustrated in Table 2, this work directly compares the modifications of previous separators with their potential future improvements.

## 4. Conclusions

To the best of our knowledge, and to distinguish this work from previous separator modification efforts that primarily focused on porosity, electrolyte compatibility, and safety issues, “electroactive” carbazole polymers are introduced in separator modification for the first time in an aim to improve full cells’ cycling performance through interphase engineering.

Separators coated with PCP and PVC have demonstrated significant increases in specific capacities, capacity retention levels, lithium inventory, and CEs in LMR-NM//Gr full cells, potentially contributing to the development of next-generation LIBs featuring high energy density and sustainability. Notably for PVC, due to its structural similarity to polyolefins and its demonstrated capability in enhancing cells’ performance with limited modification, it’s reasonably believed that it has high potential for seamless integration into the current separator manufacturing process.

Note that the LMR-NM material performs well with optimized engineering in terms of the loading of the cathodes and the composition of the slurry mixes to optimize the materials with a minimum initial impedance and impedance increase in the beginning and after cycling, respectively, so any improvement is not trivial. Similar scenarios were encountered for LiNi_0.90_Mn_0.05_Co_0.05_O_2_ [60], where performance improvement is especially difficult to realize due to the already high-performance levels. The hypothesis we have developed is that a redox-active polymer can provide the first-charge “sacrificial” oxidation for extra Li inventory and facilitate interphase engineering to boost performance. Indeed, in LMR-NM//Gr whole cells, the capacity retention rates of PCP with a 100-μm coating thickness and PVC with a 50-μm coating thickness increased from 78.13% to 87.48% and 90.14%, respectively, over that of the baseline cells. These achievements can only be ascribed to the electrochemical activity of the consequent PCP and PVC polymers, as demonstrated through our experiments using CV, NMR, dQ/dV, and galvanostatic cycling.

Based on the working mechanism revealed in this study, other carbazole polymers or polymers may also be able to improve the electrochemical performance of lithium-ion batteries and beyond for various cathode materials. Additionally, their application as cathode electrode binders or additives also has intriguing possibilities and is worth exploration.

## Figures and Tables

**Table 1:** Comparison of first cycle discharge specific capacity, 8th cycle coulombic efficiency, first cycle discharge specific capacity, initial ASI, and final ASI between baseline separator and different coatings.

Separator Used	First Cycle Discharge Specific Capacity (mAh g^−1^)	Eighth Cycle Coulombic Efficiency (%)	Final Cycle Discharge Specific Capacity (mAh g^−1^)	Initial ASI (Ω∙cm^2^)	Final ASI (Ω∙cm^2^)
Celgard 2325	106	99.03	113	37.63	137.38
PCP 50 μm coating	103.9	99.07	109	74.37	153.5
PCP 100 μm coating	103.4	98.8	98.7	92.84	174.23
PVC 50 μm coating	111.3	97.1	113.4	52.53	76.36
PVC 100 μm coating	107.4	99.2	141.4	80.87	143.66

**Table 2:** Comparison of separators and their properties, mechanisms for improvement, as well as future directions.

Properties	Mechanisms	Future Directions	References
Thermal Stability	Improved thermal stability reduces risk of thermal runaway	High-performance thermally stable separators (e.g., ceramic-coated) increase cost	[53,54,55]
Mechanical Strength	Enhanced mechanical strength prevents internal short circuits	Thick or reinforced separators can reduce ionic conductivity, lowering power density	[56]
Electrolyte Wettability	Improved wettability ensures better electrolyte uptake, enhancing ionic conductivity	Some coatings to improve wettability may degrade over cycling or cause side reactions	[55,57]
Chemical Stability	Stability against high-voltage cathodes (>4.3 V) reduces side reactions and capacity fade	Some separators degrade under high voltage or react with electrolyte additives	[58]
Ionic Conductivity	Thin separators with porous structures enable high ionic conductivity	Too thin separators may have lower puncture resistance, risking safety	[59]
Electrochemical and interfacial stability	Electroactive carbazole polymers demonstrate first cycling lithium inventory improvement and interfacial stability	Polymer may need engineering to get a thin layer	This work

## Data Availability

The original contributions presented in this study are included in the article/Supplementary Material. Further inquiries can be directed to the corresponding author.

## Supplementary Materials

The following supporting information can be downloaded at: https://www.mdpi.com/article/10.3390/polym17131815/s1, Figure S1. FTIR spectra of reactant 1 of 9-Phenyl-3,6-bis(4,4,5,5-tetramethyl-1,3,2-dioxaborolan-2-yl)-9H-carbazole (black), reactant 2 of 1,4-dibromobenzene (red), and the synthesized PCP (blue). The disappearance of characteristic B-O and C-Br vibrational bands from reactant 1 and reactant 2, respectively, in the synthesized PCP confirms its successful synthesis. Figure S2. Illustrations of PCP modified separator with a slurry coating method. Figure S3. Area specific impedance (ASI) for LMR-NM//Gr cells with varying separator coating thicknesses of PVC compared to a baseline cell using Celgard 2325. Figure S4. 1H NMR spectra of Gen2 electrolytes after stirring vigorously with plenty of (a) PCP, and (b) PVC for 2 days. Only peaks of ethylene carbonate, and ethyl methyl carbonate from Gen2 were observed. Figure S5. Differential capacity (dQ/dV) curves for the PCP 100 μm coated separator cell compared to the baseline cell: (a) cycle 2, the second formation cycle; (b) cycle 4, the first activation cycle; and (c) cycle 8, the first C/3 aging cycle. Figure S6. Differential capacity (dQ/dV) curves for the PCP 50 μm coated separator cell compared to the baseline cell: (a) cycle 1, the first formation cycle; (b) cycle 2, the second formation cycle; (c) cycle 4, the first activation cycle; and (d) cycle 8, the first C/3 aging cycle. Figure S7. Differential capacity (dQ/dV) curves for the PCP 150 μm coated separator cell compared to the baseline cell: (a) cycle 1, the first formation cycle; (b) cycle 2, the second formation cycle; (c) cycle 4, the first activation cycle; and (d) cycle 8, the first C/3 aging cycle. Figure S8. Charge specific capacities for LMR-NM//Gr full cells using PCP modified Celgard 2325 separators with different coating thicknesses compared to the cell using baseline Celgard 2325 separator. Figure S9. Cyclic voltammetry curves of PCP//Cu cell compared to Al//Cu cell at a scan rate of 0.1mV/s. Figure S10. Illustration of the insolubility of (a) PCP, and the solubility of (b) oxidized PCP in DMSO-d6. Figure S11. N1s, Li1s, Mn2p, Mn3p, and Ni2p regions of XPS spectra for the anodes collected from cycled PCP coating cell compared to the baseline. Figure S12. N1s, Li1s, Mn2p, and Ni2p regions of XPS spectra for the cathodes collected from cycled PCP coating cell compared to the baseline. Figure S13. (a) Coulombic efficiencies, and (b) charge specific capacities of the initial cycles for LMR-NM//Gr full cells using PVC modified Celgard 2325 separators with different coating thicknesses compared to the cell using baseline Celgard 2325 separator. The inserted numbers are the initial Coulombic efficiencies for each cell as denoted. Figure S14. Differential capacity (dQ/dV) curves for the PVC 50 μm coated separator cell compared to the baseline cell: (a) cycle 1, the first formation cycle; (b) cycle 2, the second formation cycle; (c) cycle 4, the first activation cycle; and (d) cycle 8, the first C/3 aging cycle. Figure S15. Differential capacity (dQ/dV) curves for the PVC 100 μm coated separator cell compared to the baseline cell: (a) cycle 1, the first formation cycle; (b) cycle 2, the second formation cycle; (c) cycle 4, the first activation cycle; and (d) cycle 8, the first C/3 aging cycle. Table S1. The initial, final, and percentage increase of ASI values for each PCP cell and the baseline. The initial and final ASI values are obtained at the lowest impedance points. Table S2. The initial, final, and percentage increase of ASI values for each PVC cell and the baseline. The initial and final ASI values are obtained at the lowest impedance points. Table S3. Electrolyte uptake of different separators calculated by immersing separators into Gen2 electrolyte.
