# Supplementary material for: Interphase Engineering Enabled by Using a Separator with Electrochemically Active Carbazole Polymers for Lithium-Ion Batteries
Source: Polymers (Basel). 2025 Jun 29;17(13):1815. doi: 10.3390/polym17131815 (PMC12252323; doi:10.3390/polym17131815)
Supplement: Supplementary file 1 [file polymers-17-01815-s001.zip › polymers-3698380-supplementary.pdf]

Supporting Information (SI)

Interphase Engineering Enabled by Separator with Electrochemical Active Carbazole Polymers for Lithium-ion Batteries

Bingning Wang,[1,2] Lihong Gao,[1] Zhenzhen Yang,[1] Xianyang Wu,[1] Qijia Zhu,[1] Qian Liu,[1] Fulya Dogan,[1] Zhengcheng Zhang,[1] Yang Qin,[1] Chen Liao[1,3]

[1] Chemical Sciences and Engineering Division, Argonne National Laboratory, 9700 South Cass Avenue, Lemont, Illinois, 60439, United States of America

[2] Department of Chemical & Biomolecular Engineering, Institute of Material Science, University of Connecticut, University of Connecticut, Storrs, Connecticut, 06269, United States of America

[3] Energy Storage Research Alliance, Argonne National Laboratory, 9700 South Cass Avenue, Lemont, Illinois, 60439, United States of America

Corresponding author: Chen Liao, liaoc@anl.gov

Table of Contents

Figures……………………………………………………………………………...…..3

Tables……………………………………………………………………………...…....14

Figures

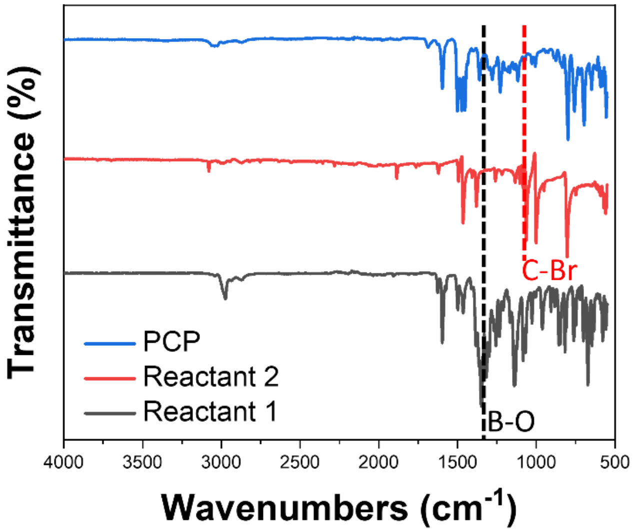

Figure S1. FTIR spectra of reactant 1 of 9-Phenyl-3,6-bis(4,4,5,5-tetramethyl-1,3,2-dioxaborolan-2-yl)-9H-carbazole (black), reactant 2 of 1,4-dibromobenzene (red), and the synthesized PCP (blue). The disappearance of characteristic B-O and C-Br vibrational bands from reactant 1 and reactant 2, respectively, in the synthesized PCP confirms its successful synthesis.

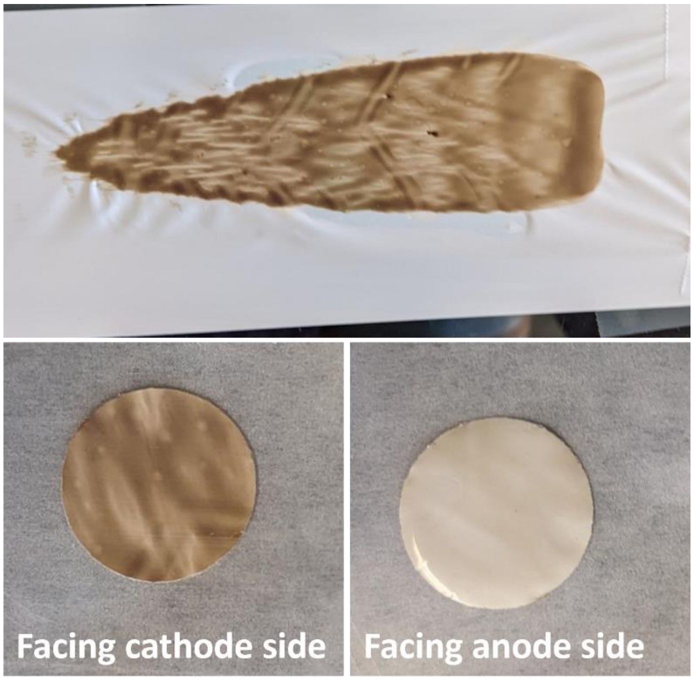

Figure S2. Illustrations of PCP modified separator with a slurry coating method.

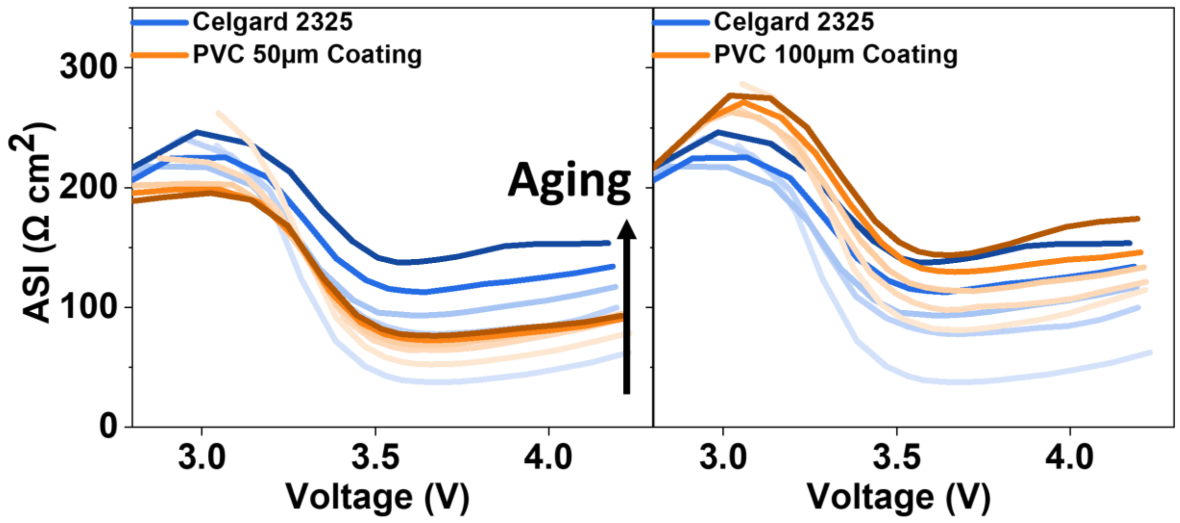

Figure S3. Area specific impedance (ASI) for LMR-NM//Gr cells with varying separator coating thicknesses of PVC compared to a baseline cell using Celgard 2325.

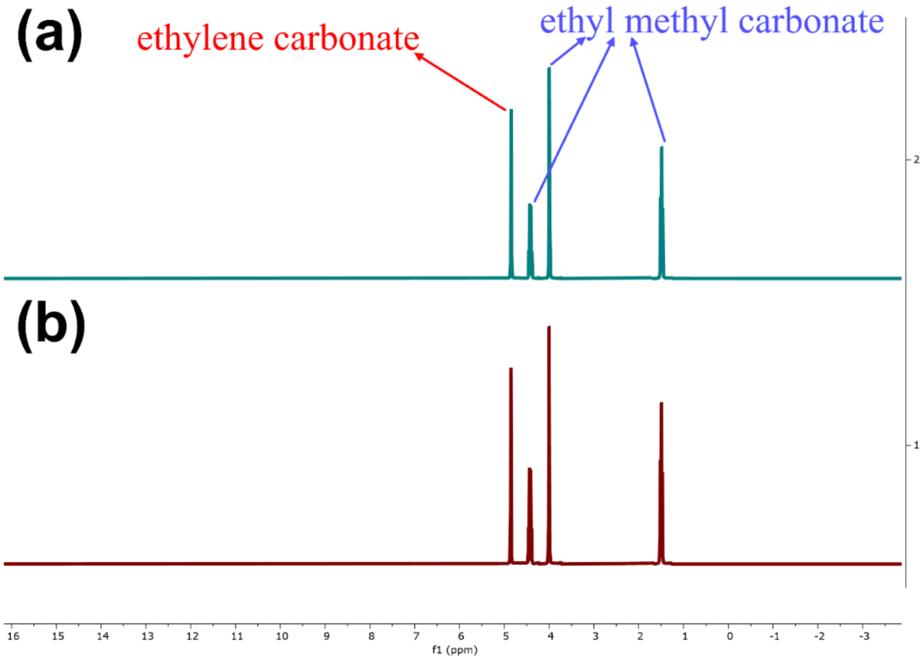

Figure S4. ^{1}H NMR spectra of Gen2 electrolytes after stirring vigorously with plenty of (a) PCP, and (b) PVC for 2 days. Only peaks of ethylene carbonate, and ethyl methyl carbonate from Gen2 were observed.

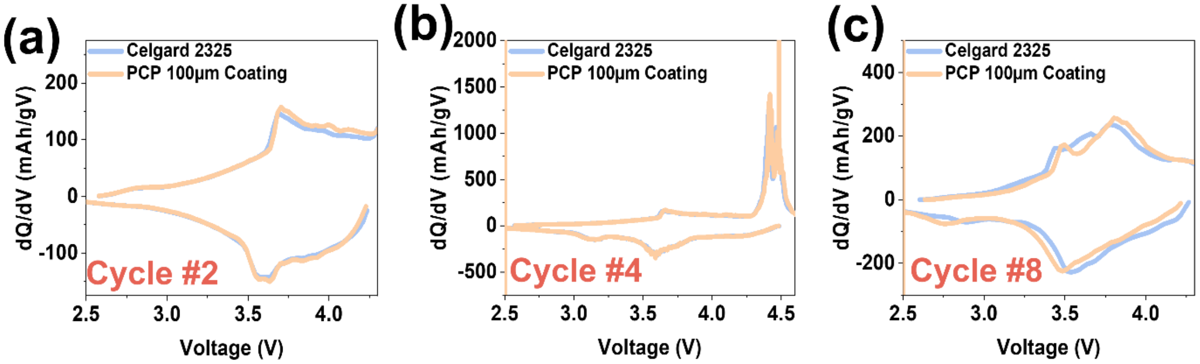

Figure S5. Differential capacity (dQ/dV) curves for the PCP 100 μm coated separator cell compared to the baseline cell: (a) cycle 2, the second formation cycle; (b) cycle 4, the first activation cycle; and (c) cycle 8, the first C/3 aging cycle.

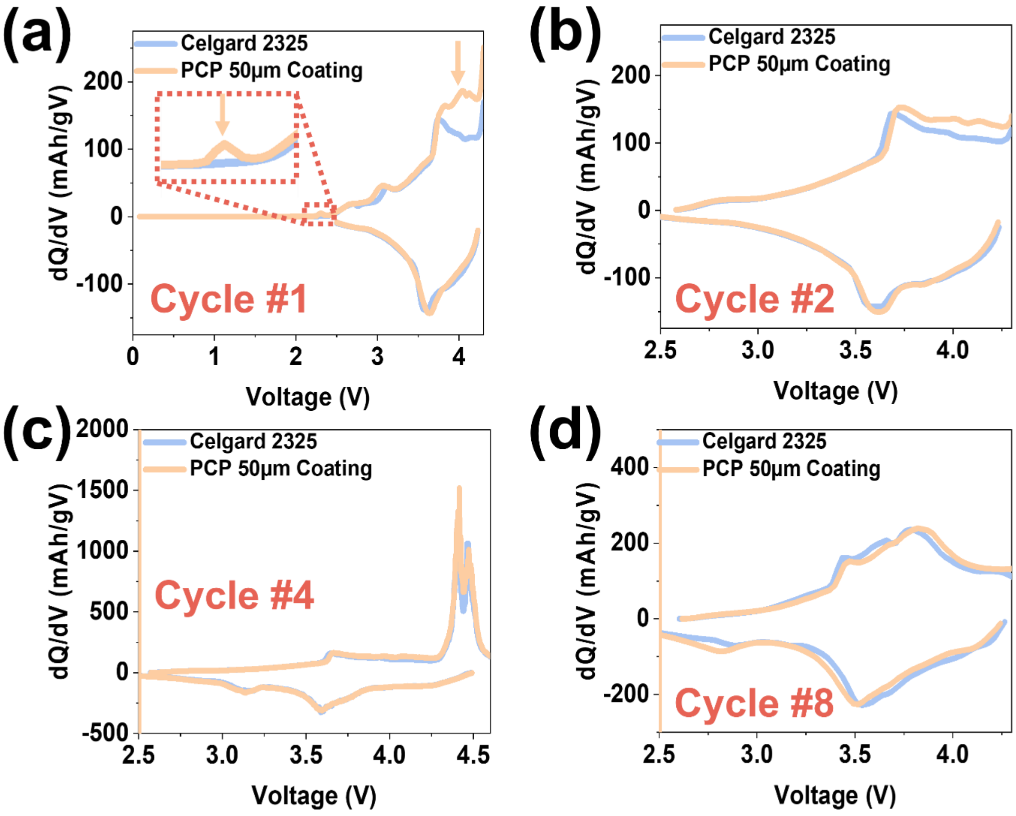

Figure S6. Differential capacity (dQ/dV) curves for the PCP 50 μm coated separator cell compared to the baseline cell: (a) cycle 1, the first formation cycle; (b) cycle 2, the second formation cycle; (c) cycle 4, the first activation cycle; and (d) cycle 8, the first C/3 aging cycle.

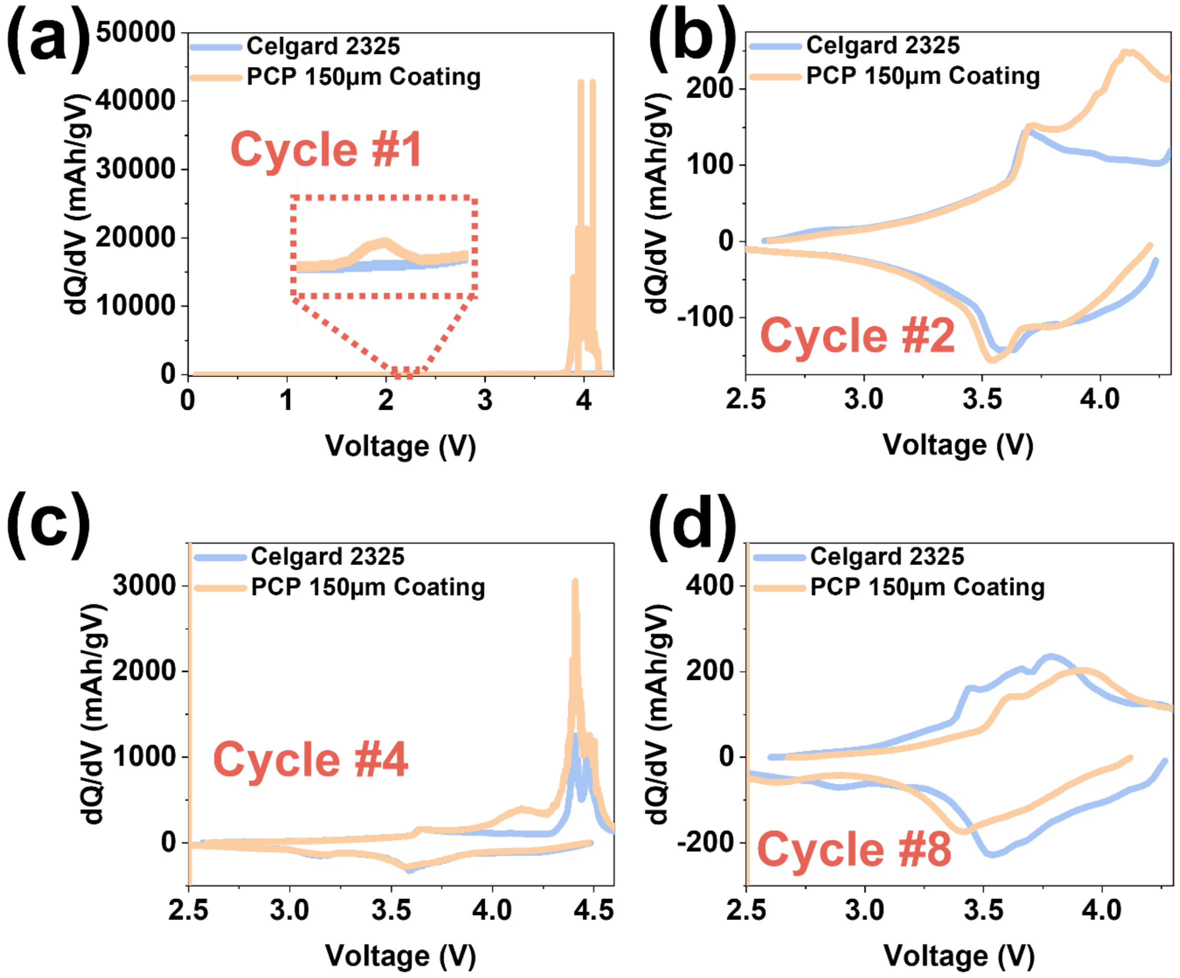

Figure S7. Differential capacity (dQ/dV) curves for the PCP 150 μm coated separator cell compared to the baseline cell: (a) cycle 1, the first formation cycle; (b) cycle 2, the second formation cycle; (c) cycle 4, the first activation cycle; and (d) cycle 8, the first C/3 aging cycle.

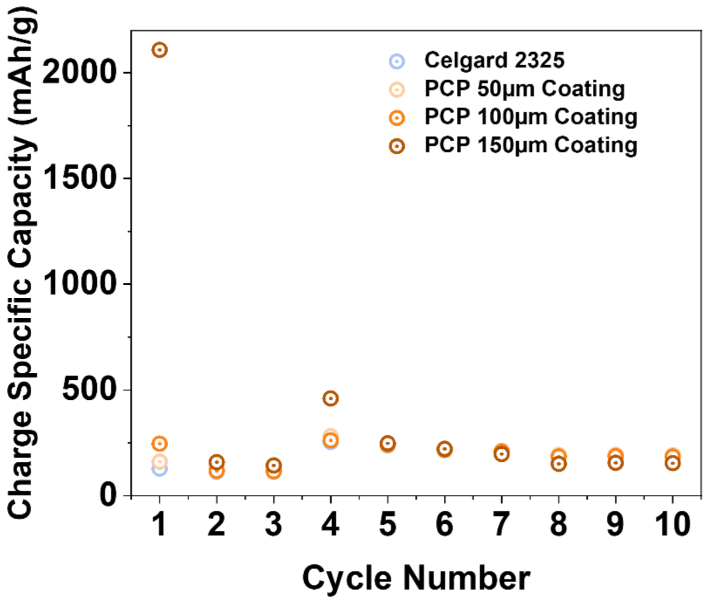

Figure S8. Charge specific capacities for LMR-NM//Gr full cells using PCP modified Celgard 2325 separators with different coating thicknesses compared to the cell using baseline Celgard 2325 separator.

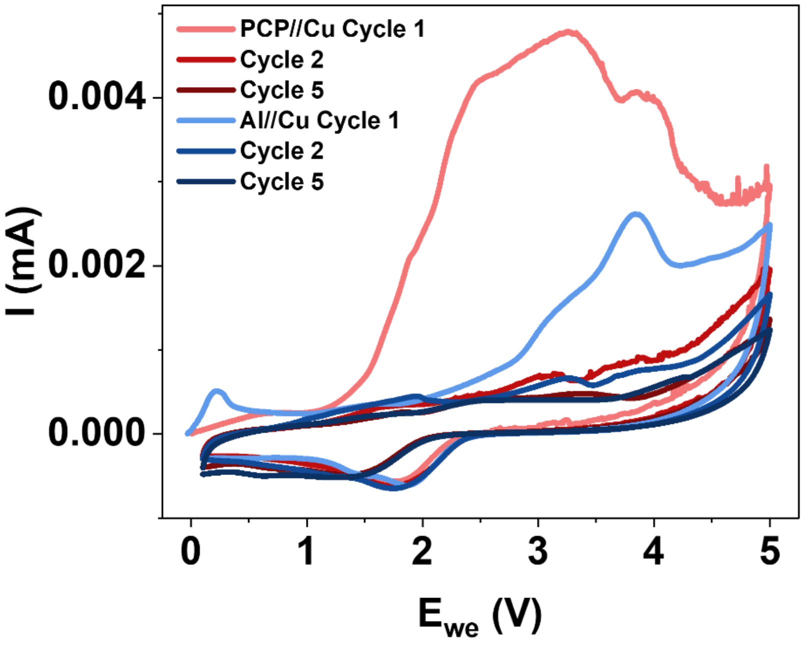

Figure S9. Cyclic voltammetry curves of PCP//Cu cell compared to Al//Cu cell at a scan rate of 0.1mV/s.

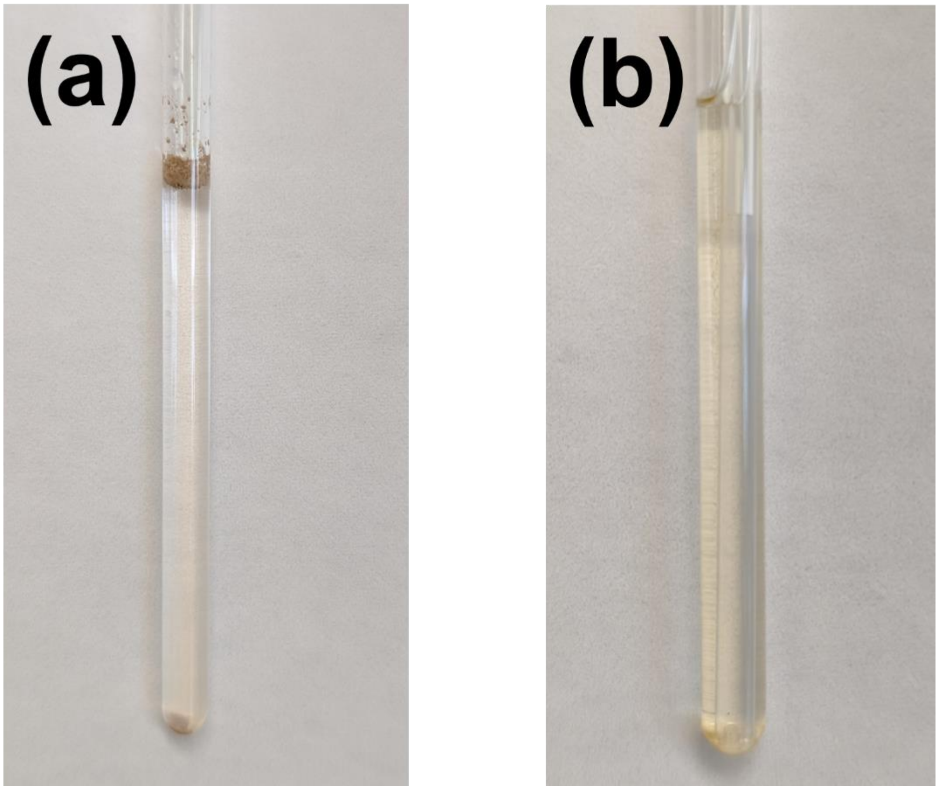

Figure S10. Illustration of the insolubility of (a) PCP, and the solubility of (b) oxidized PCP in DMSO-d_6.

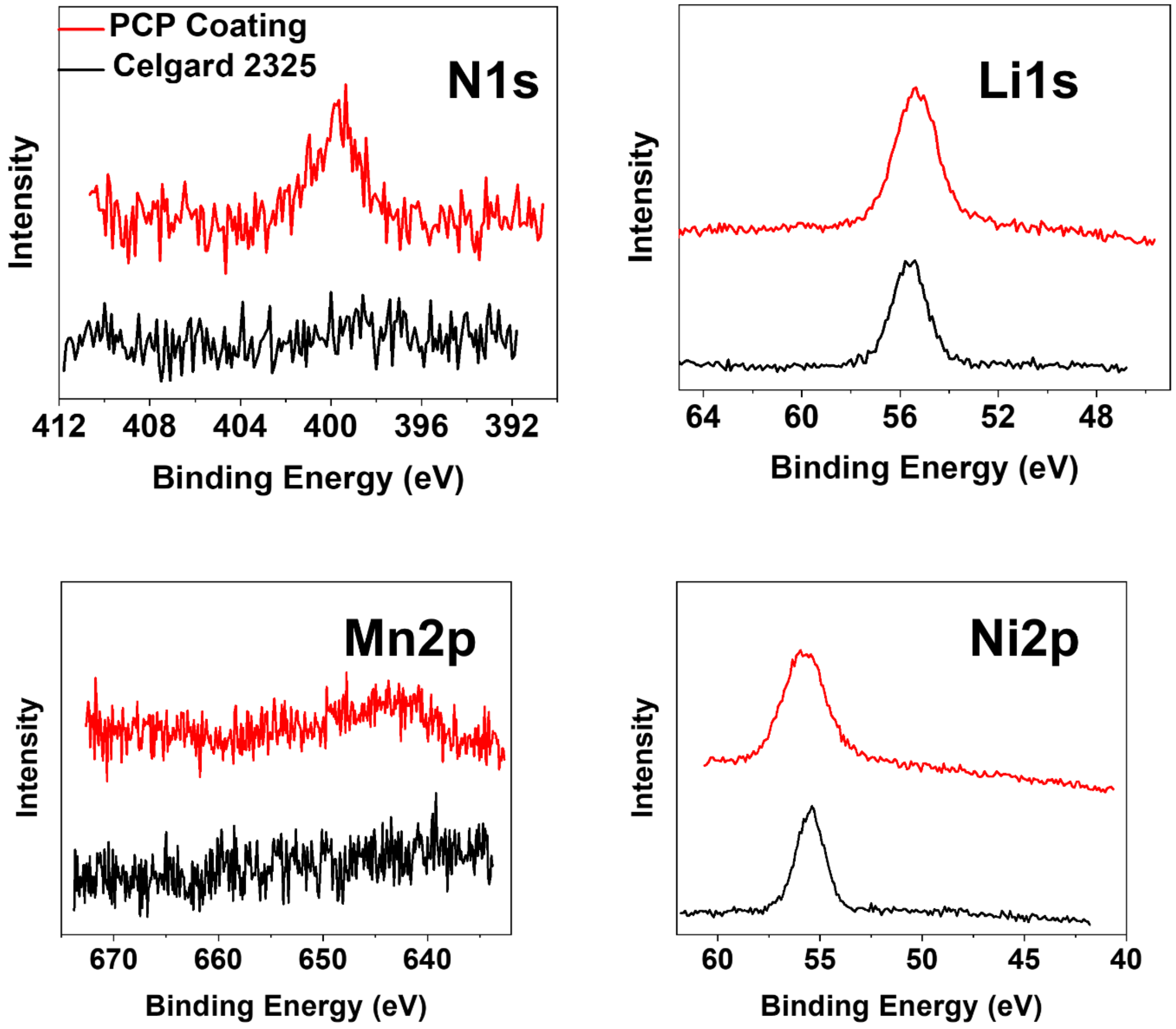

Figure S11. N1s, Li1s, Mn2p, Mn3p, and Ni2p regions of XPS spectra for the anodes collected from cycled PCP coating cell compared to the baseline.

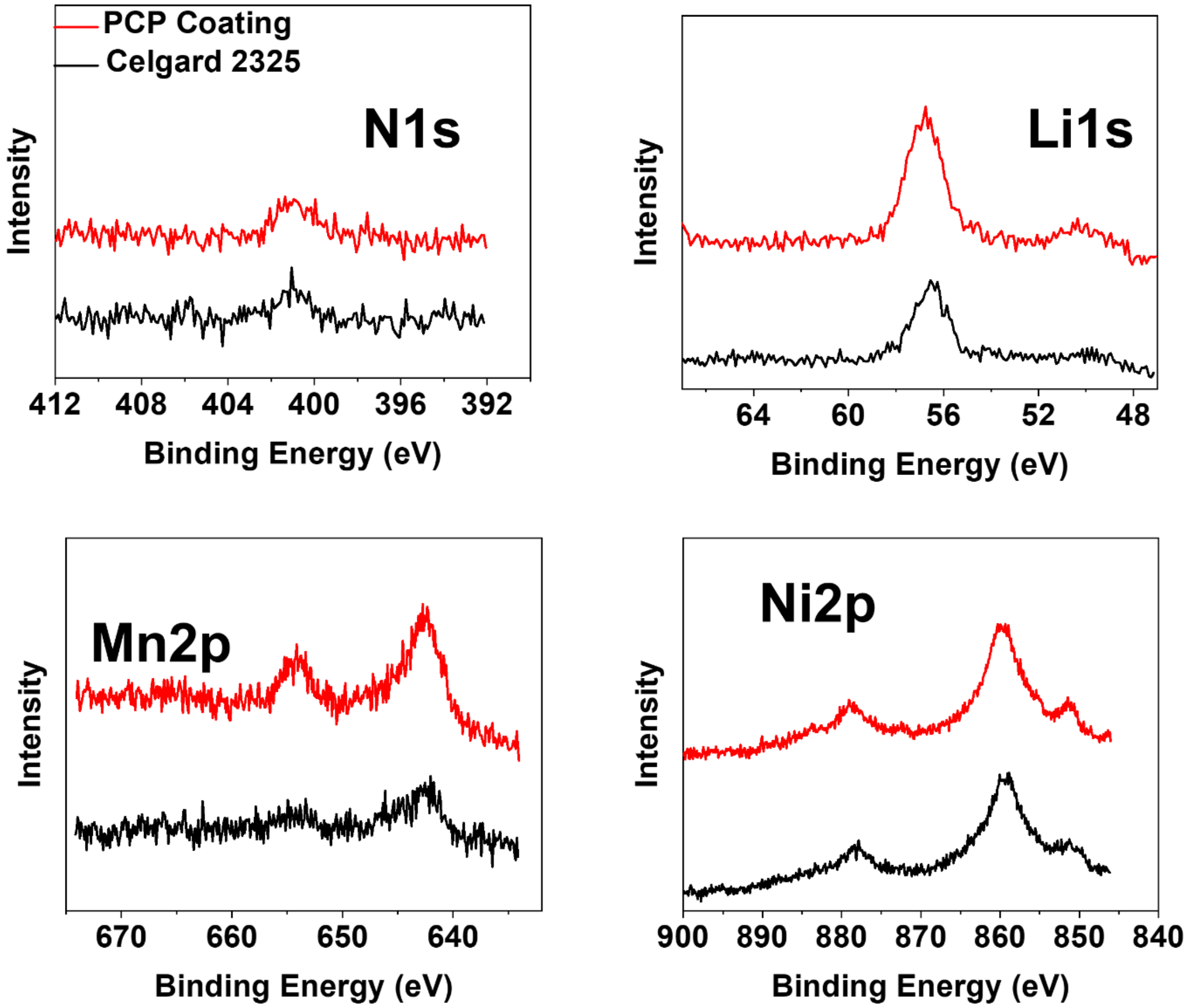

Figure S12. N1s, Li1s, Mn2p, and Ni2p regions of XPS spectra for the cathodes collected from cycled PCP coating cell compared to the baseline.

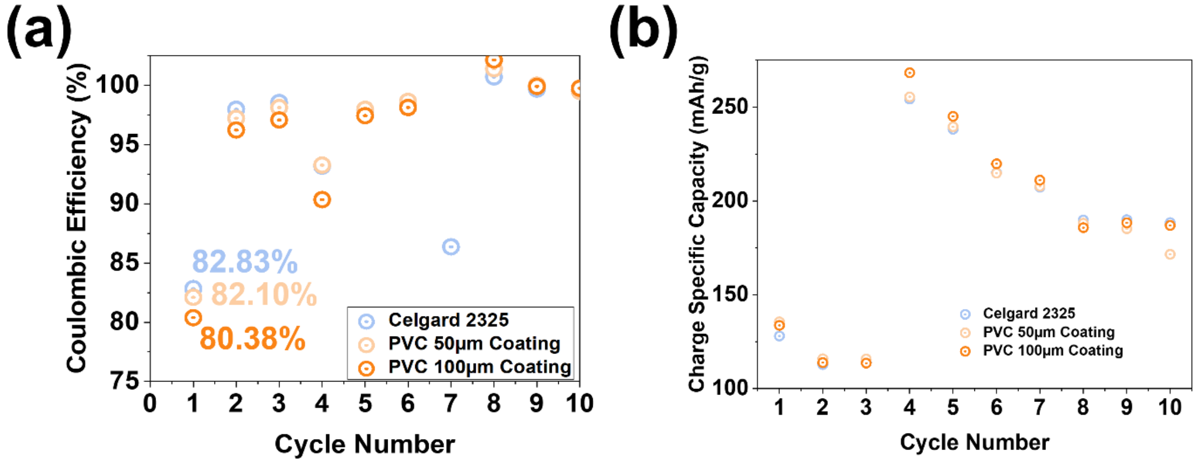

Figure S13. (a) Coulombic efficiencies, and (b) charge specific capacities of the initial cycles for LMR-NM//Gr full cells using PVC modified Celgard 2325 separators with different coating thicknesses compared to the cell using baseline Celgard 2325 separator. The inserted numbers are the initial Coulombic efficiencies for each cell as denoted.

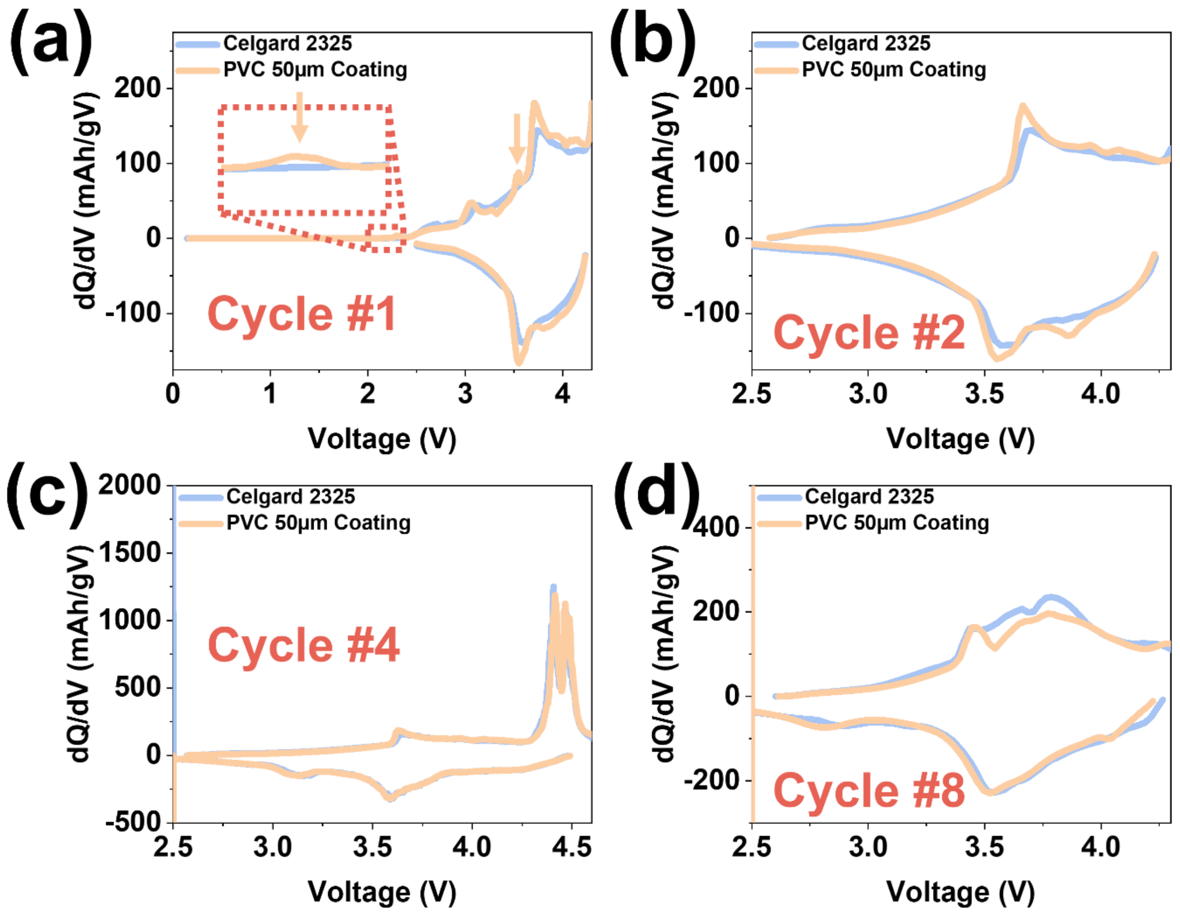

Figure S14. Differential capacity (dQ/dV) curves for the PVC 50 μm coated separator cell compared to the baseline cell: (a) cycle 1, the first formation cycle; (b) cycle 2, the second formation cycle; (c) cycle 4, the first activation cycle; and (d) cycle 8, the first C/3 aging cycle.

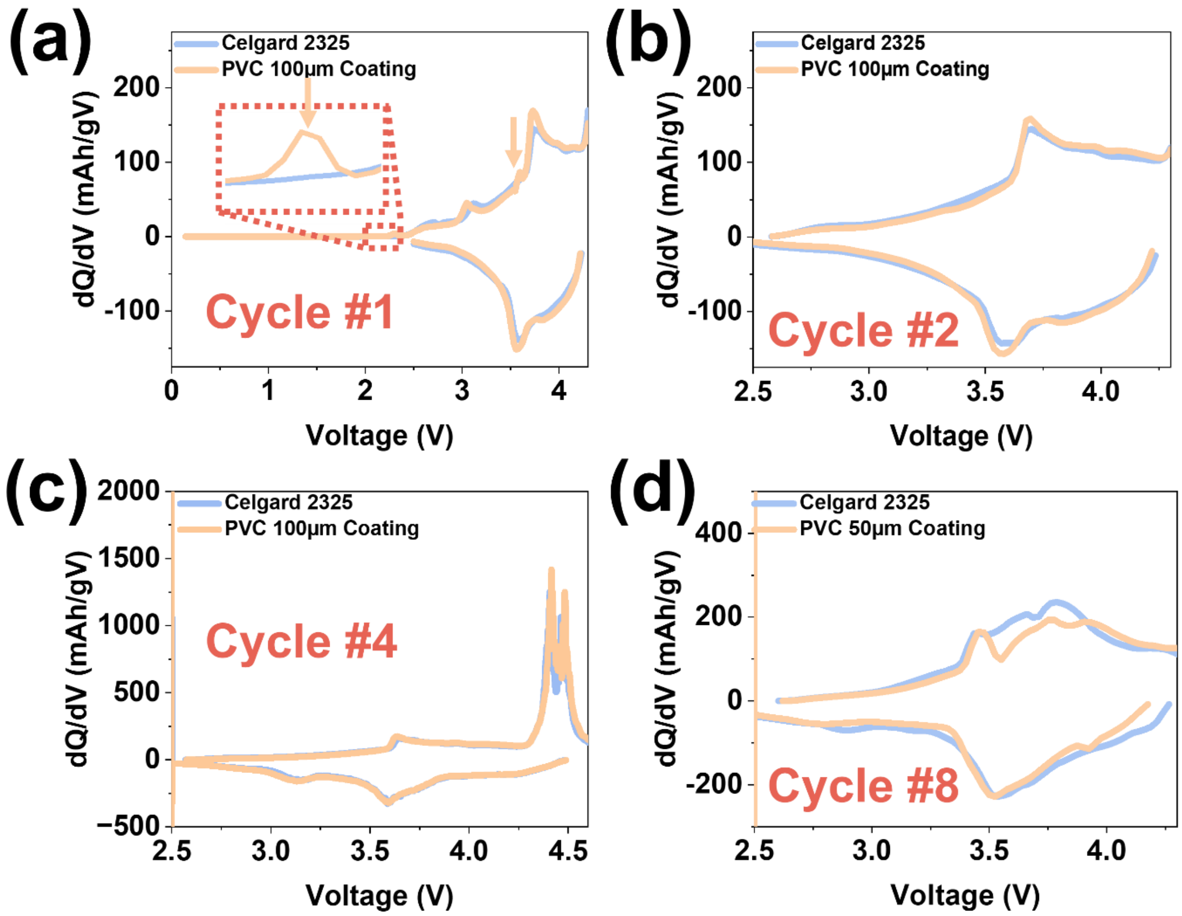

Figure S15. Differential capacity (dQ/dV) curves for the PVC 100 μm coated separator cell compared to the baseline cell: (a) cycle 1, the first formation cycle; (b) cycle 2, the second formation cycle; (c) cycle 4, the first activation cycle; and (d) cycle 8, the first C/3 aging cycle.

Tables

ASI Impedance (Ω cm^2)	Celgard 2325 Baseline	PCP 50µm Coating	PCP 100µm Coating	PCP 150µm Coating
Initial	37.63	74.37	92.84	170.97
Final	137.38	153.50	174.23	222.37
% increase	265.08%	106.48%	87.67%	30.01%

Table S1. The initial, final, and percentage increase of ASI values for each PCP cell and the baseline. The initial and final ASI values are obtained at the lowest impedance points.

ASI Impedance (Ω cm2)	Celgard 2325 Baseline	PVC 50µm Coating	PVC 100µm Coating
Initial	37.63	52.53	80.87
Final	137.38	76.36	143.66
% increase	265.08%	45.36%	77.64%

Table S2. The initial, final, and percentage increase of ASI values for each PVC cell and the baseline. The initial and final ASI values are obtained at the lowest impedance points.

	Weight before Immersing in Gen2 (mg)	Weight of the Coating	Weight after Immersing in Gen2 (mg)	Electrolyte Uptake
Celgard 2325	2.99	0	5.24	75.25%
Celgard 2325_PCP 50 µm coating	3.47	0.48	5.28	52.16%
Celgard 2325_PCP 100 µm coating	4.04	1.05	5.89	45.79%
Celgard 2325_PVC 50 µm coating	3.87	0.88	5.43	40.31%
Celgard 2325_PVC 100 µm coating	4.74	1.75	6.10	28.69%

Table S3. Electrolyte uptake of different separators calculated by immersing separators into Gen2 electrolyte.
